# Increasing prevalence of breast cancer among Saudi patients attending a tertiary referral hospital: a retrospective epidemiologic study

**DOI:** 10.3325/cmj.2012.53.239

**Published:** 2012-06

**Authors:** Ammar Al-Rikabi, Sufia Husain

**Affiliations:** Department of Pathology, College of Medicine, King Saud University, Riyadh, Saudi Arabia

## Abstract

**Aim:**

To determine the pattern of breast diseases among Saudi patients who underwent breast biopsy, with special emphasis on breast carcinoma.

**Methods:**

A retrospective review was made of all breast biopsy reports of a mass or lump from male and female patients seen between January 2001 and December 2010 at the King Khalid University Hospital, Riyadh, Saudi Arabia.

**Results:**

Of 1035 breast tissues reviewed, 939 specimens (90.7%) were from female patients. There were 690 benign (65.8%) and 345 (34.2%) malignant cases. In women, 603 (64.2%) specimens were benign and 336 (35.8%) were malignant. In men, 87 specimens (90.6%) were benign and 9 (9.4%) were malignant. All malignant cases from male patients belonged to invasive ductal carcinoma and the majority of malignant cases from female patients belonged to invasive/infiltrating ductal carcinoma. The proportion of malignancy was 18% in patients younger than 40 years and 63.2% in patients older than 60 years. The mean age of onset for malignancy was 48.6 years. The annual percentage incidence of malignant breast cancer steadily increased by 4.8%, from an annual rate of 23.5% in 2000 to 47.2% in 2007.

**Conclusion:**

Among Saudi patients, there is a significant increase in the incidence of breast cancer, which occurs at an earlier age than in western countries. Continued vigilance, mammographic screening, and patient education are needed to establish early diagnosis and perform optimal treatment.

Increased awareness and efficient breast cancer information-dissemination campaign led to an increased number of diagnosed cases of breast cancer. According to the American Cancer Society, about 1.3 million American women annually are diagnosed with breast cancer and about 465 000 die from the disease (1). The number of deaths has decreased since 1990, probably due to an earlier detection and advances in treatment. According to 2000-2004 Saudi National Cancer Registry data, there were 127.8 per 100 000 women with breast cancer and mortality rate was 25.5 per 100 000 (2).

Most palpable breast masses are benign; less than 30% of women with palpable masses have a diagnosis of cancer (3-5). Approximately 4% of breast cancers present with a palpable mass without mammographic or ultrasonographic evidence of the disease (6). Therefore, evaluation of a breast mass should be done by taking into consideration patient’s history, physical examination, imaging, and biopsy. Definitive diagnosis in nearly all cases is established by needle biopsy. Because of the low specificity of mammography, many women undergo unnecessary breast biopsy. As many as 65%-85% of breast biopsies are performed on benign lesions (7), which subjects the patients to avoidable emotional and physical burden.

Similarly to other countries, breast cancer in Saudi Arabia is the most common cancer in women (7). The Saudi National Cancer Registry reported a rising proportion of breast cancer among women of all ages, from 10.2% in 2000 to 24.3% in 2005 (8). A significant majority of these breast cancers (almost 80%) were of the infiltrating ductal type. The average age at presentation of breast cancer in Arab countries is 48 years, which is a decade earlier than in western countries (9). The median age of onset of breast cancer among Saudi women is 46 years (8). Due to the increasing incidence, several articles have been published on screening for breast cancer and on public awareness programs initiated by the Saudi Arabian government and non-governmental sectors (10-13). This study aims to describe the epidemiological characteristics of breast mass lesions of patients examined at the King Khalid University Hospital, Riyadh, Saudi Arabia from 2001 to 2010.

## Materials and methods

This retrospective study included histopathological reports of all patients (including male patients) who had undergone biopsy of breast masses or lesions between January 2001 and December 2010 at the King Khalid University Hospital, which is a large (1000 beds) tertiary referral hospital with a large patients catchment area covering the northern part of Riyadh. Data were extracted from the hospital computer database and entered into an Excel sheet. Demographic data included age at diagnosis and sex. Histomorphological features were classified as benign or malignant based on the 2003 World Health Organization classification of tumors of the breast (14). Data were analyzed using Predictive Analytical Software, version 18 (IBM, SPSS Inc., Chicago, IL, USA) and expressed as mean, standard deviation, and percentages. Validation was performed by random re-examination by the two authors of 15% of the histopathology slides representing the various breast lesions.

## Results

A total of 1035 breast tissue specimens were examined between January 2001 and December 2010 at the Pathology Department of the King Khalid University Hospital. There were 939 specimens (90.7%) from female and 96 (9.3%) from male patients. Histopathology revealed 681 (65.8%) benign and 354 (34.2%) malignant cases. The average age at diagnosis of all patients with malignancy was 48.6 ± 11.6 years.

Among men, benign breast lesions mostly occurred in patients younger than 40 years, while malignant lesions occurred in patients aged 40 and older (Table 1). Among women, benign breast lesions also mostly occurred in patients younger than 40 years and the majority of those cases (more than 50%) were fibroadenomas. The median age of onset of breast cancer in our population was 48.0 years (range, 20-91 years). However, the proportion of malignancy progressively increased from 18% in the age group 20-39 years to 53.3% in the age group 40-59 years, and as much as 63.2% of patients were more than 60 years old (Table 2).

**Table 1:** Breast lesions detected in male patients with age distribution

	Age groups in years, n (%)				
	<20	20-39	40-59	60 and above	Total
Benign (n = 87):					
gynecomastia	18 (90.0)	53 (88.3)	5 (50)	-	76 (79.2)
fibroadenoma	1 (5.0)	3 (5)	1 (10)	-	5 (5.2)
lipoma	1 (5.0)	4 (6.7)	-	-	5 (5.2)
trichoadenoma				1 (16.7)	1 (1.0)
Malignant (n = 9):					
invasive ductal carcinoma	-	-	4 (40)	5 (83.3)	9 (9.4)
Total	20	60	10	6	96

**Table 2:** Breast lesions seen in female breasts with age distribution

	Age groups in years, n (%)				
	<20	20-39	40-59	60 and above	Total
Benign (n = 603):					
fibroadenoma	57 (85.1)	218 (56.3)	132 (31.7)	11 (16.2)	418 (69.3)
normal	5 (7.5)	32 (8.3)	26 (6.2)	2 (2.9)	65 (10.8)
duct ectasia	-	14 (3.6)	7 (1.7)	5 (7.4)	26 (4.3)
granulomatous mastitis	1 (1.5)	13 (3.4)	10 (2.4)	1 (1.5)	25 (4.1)
abscess	-	12 (3.1)	4 (0.9)		16 (2.7)
lipoma	3 (4.5)	7 (1.8)	4 (0.9)	2 (2.9)	16 (2.7)
intraductal papilloma	1 (1.5)	3 (0.8)	7 (1.7)	3 (4.4)	14 (2.3)
fat necrosis	-	8 (2.1)	2 (0.5)	1 (1.5)	11 (1.8)
epidermal inclusion cyst	-	3(0.8)	1 (0.2)	-	4 (0.7)
galactocoele	-	4 (1.0)	-	-	4 (0.7)
benign phyllodes tumor	-	3(0.8)	1 (0.2)	-	4 (0.7)
Malignant (n = 336):					
invasive ductal carcinoma	-	66 (17.0)	217 (52.0)	40 (58.8)	323 (96.1)
malignant phyllodes tumor	-	2 (0.5)	3 (0.7)	1 (1.5)	6 (1.8)
medullary breast cancer	-	2 (0.5)	1 (0.2)	-	3 (0.9)
mucinous colloid carcinoma	-	-	1 (0.2)	2 (2.9)	3 (0.9)
inflammatory carcinoma	-	-	1 (0.2)	-	1 (0.3)
Total	67 (7.1)	387 (41.2)	417 (44.4)	68 (7.2)	939

**Figure 1:**
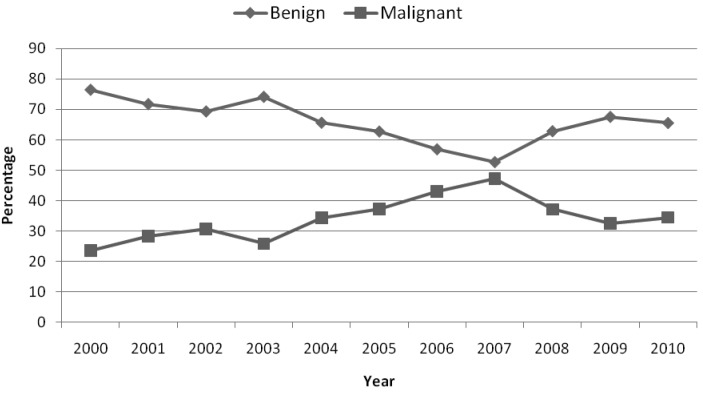
Annual percentage and distribution of benign vs malignant cases of breast lesions over a period of ten years. Rhomb – benign; square – malignant.

The yearly percentage incidence of malignant breast neoplasms (in terms of percentage distribution) showed a steady rise over the years, as compared to benign breast lesions. In 2000, 76.5% of the tissues submitted for histopathology were benign and only 23.5% were malignant. From 2000 to 2007, there was a steady rise by a mean of 4.8% in the annual incidence of malignant breast lesions, with the exception of 2003, when there was a slight decrease. The annual rate of a malignant breast lesions confirmed by histopathological examination ranged from 23.5% in 2000 to 47.2% in 2007. In contrast, after 2007, there was a shift of the trend toward more benign cases (Figure 1). There was, however, an overall increase in the percentage of people diagnosed with breast cancer from 23.5% in 2000 to 34.5% in 2010.

## Discussion

In this study, the majority of breast lesions sent for histopathology were fibroadenomas, constituting 423 of 1035 (40.9%) breast specimens received in our laboratory. This finding is similar to other reports (1,2). Such patients were predominantly young, with age range from 18 to 78 years in women. Although fibroadenomas are not cancerous or life-threatening lesions they can still be a source of significant anxiety and concern to the patient.

Our study found a malignancy rate of 33.3% (ie, 345 out of 1035 cases were malignant). It is a high rate, compared to the rate reported in the literature that was lower than 30% (3-5). Furthermore, our incidence rate was significantly higher than the incidence rate of 24.3% reported by the Saudi National Cancer Registry in 2005 (8). This difference may be explained by an increase in the use of mammography, effectiveness of breast cancer awareness program that has been initiated by the government, the effective campaign organized by various health authorities in order to educate the public on early detection of breast cancer through self breast examination, or sample bias (10-13). In our study, infiltrating/invasive ductal carcinoma was the most common malignant type of breast cancer (96.2%), with a slightly higher rate than that reported by the Saudi National Cancer Registry (8). Patients diagnosed with breast cancer were in the age groups ranging from 28 to 78 years; however most of the women were in the age group 40-59 years (97.3%) (8). The age of onset of breast cancer in our patients was similar to that previously reported (9) but was slightly higher than that reported by the Saudi National Cancer Registry (8).

Although our findings did not substantially differ from those in previous studies in terms of the age of presentation of breast cancer, histopathological types, or even the ratio of benign to malignant cases, we believe that it is necessary to point out the steady rise in the incidence of breast cancer since 2000, with a mean annual increase of 4.8%. This rate is higher than the reported annual increase in the proportion of breast cancer of 2% in the UK (15) and higher than the annual average increase of 0.4% in the USA (16). This trend can be explained either by an effective awareness program and efficient patient education on breast self-examination or by sample bias as our patients came from only one referral center. It is also important to emphasize that breast cancer remains the leading cause of death among the Saudi women.

Furthermore, we noticed a changing pattern in the proportion of breast cancer cases in the last 3 years (2007 to 2010). Before 2007, most of the breast specimens were benign cases, with an increasing annual incidence of malignant cases. However, after 2007, the trend shifted toward benign cases. In our opinion, this is a reflection of an effective campaign of breast diseases awareness programs. Nevertheless, this trend should not be a reason for complacency, since some of these benign cases, though not cancerous, may indicate an increased risk toward the development of pre-malignant conditions such as fibroadenomas (17). Another point of paramount importance, highlighted in this and other studies (9,15), is that among Saudi patients breast carcinoma occurs in relatively younger age groups than is the case in Western patients. This could be due to the demography of the Saudi population, which is characterized by a predominance of a younger population (more than 60% of the population is under 18 years) (8).

An important limitation of this study is its retrospective nature and, although our study covered a large catchment area, all the data were from one tertiary referral hospital, which is why they might not be representative of the entire country. There is a need for a larger study involving various medical centers that would also examine other environmental, genetic, and dietary factors that may contribute to the incidence of breast diseases in this part of the world.
